# Prediction of Gold Nanoparticle and Microwave-Induced Hyperthermia Effects on Tumor Control via a Simulation Approach

**DOI:** 10.3390/nano9020167

**Published:** 2019-01-29

**Authors:** Nikolaos M. Dimitriou, Athanasia Pavlopoulou, Ioanna Tremi, Vassilis Kouloulias, Georgios Tsigaridas, Alexandros G. Georgakilas

**Affiliations:** 1 Department of Physics, School of Applied Mathematical and Physical Sciences, National Technical University of Athens (NTUA), Zografou Campus, 15780 Athens, Greece; nikolaos.dimitriou@mail.mcgill.ca (N.M.D.); ioannatremi@mail.ntua.gr (I.T.); gtsig@mail.ntua.gr (G.T.); 2 Department of Bioengineering, McGill University, Montreal, QC H3A 0E9, Canada; 3 Izmir International Biomedicine and Genome Institute, Dokuz Eylül University, 35340 Balcova, Turkey; athanasia.pavlopoulou@deu.edu.tr; 4 Radiation Oncology Unit, 2nd Department of Radiology, Attikon University General Hospital, Medical School, National and Kapodistrian University of Athens, 15772 Athens, Greece; vkouloul@ece.ntua.gr

**Keywords:** hyperthermia, gold nanoparticles, simulation, thermoresistance, tumor control

## Abstract

Hyperthermia acts as a powerful adjuvant to radiation therapy and chemotherapy. Recent advances show that gold nanoparticles (Au-NPs) can mediate highly localized thermal effects upon interaction with laser radiation. The purpose of the present study was to investigate via in silico simulations the mechanisms of Au-NPs and microwave-induced hyperthermia, in correlation to predictions of tumor control (biological endpoints: tumor shrinkage and cell death) after hyperthermia treatment. We also study in detail the dependence of the size, shape and structure of the gold nanoparticles on their absorption efficiency, and provide general guidelines on how one could modify the absorption spectrum of the nanoparticles in order to meet the needs of specific applications. We calculated the hyperthermia effect using two types of Au-NPs and two types of spherical tumors (prostate and melanoma) with a radius of 3 mm. The plasmon peak for the 30 nm Si-core Au-coated NPs and the 20 nm Au-NPs was found at 590 nm and 540 nm, respectively. Considering the plasmon peaks and the distribution of NPs in the tumor tissue, the induced thermal profile was estimated for different intervals of time. Predictions of hyperthermic cell death were performed by adopting a three-state mathematical model, where “three-state” includes (i) alive, (ii) vulnerable, and (iii) dead states of the cell, and it was coupled with a tumor growth model. Our proposed methodology and preliminary results could be considered as a proof-of-principle for the significance of simulating accurately the hyperthermia-based tumor control involving the immune system. We also propose a method for the optimization of treatment by overcoming thermoresistance by biological means and specifically through the targeting of the heat shock protein 90 (HSP90), which plays a critical role in the thermotolerance of cells and tissues.

## 1. Introduction

Although, great advances have been made in early diagnosis and treatment of cancer, it remains one of the leading causes of death worldwide. Radiotherapy, chemotherapy, and surgery represent the main modalities of cancer treatment, while recent advances in the biology of cancer led to the development of new therapeutic strategies that result in the attack of the cancer cells by the immune system. However, a great number of cancer patients develop side effects, thereby making these treatments painful and very unpleasant due to the intrinsic sensitivity of the adjacent normal tissue [1], while novel therapies such as immunotherapy are still very expensive and inefficient [2]. As a result, targeted therapies are becoming increasingly urgent because they can minimize any side effects and make the treatment more efficient. Even though hyperthermia is not the preferred method of primary cancer treatment, historical evidence suggests that thermal therapy was used in ancient times; as Hippocrates stated “*what medicines do not heal, the lance will; what the lance does not heal, fire will*” [3]. Hyperthermia, with or without enhancement of its effects by gold nanoparticles (Au-NPs), has the potential of eliminating the side toxic effects of traditional cancer therapies acting primarily as an adjuvant to radiation or chemotherapy [4]. Metallic nanoparticles (NPs), like gold NPs, strongly absorb and scatter light close to their localized surface plasmon resonance (LSPR) and therefore can be used as heat emitters. As a result, they convert electromagnetic energy into heat, thereby causing hyperthermic cellular damage [5]. Heat delivered within the hyperthermia range (42–48 °C) induces tumor cell death, primarily by denaturation of essential cellular proteins [6].

The efficacy of LSPR in gold nanoparticles changes among different shapes [7,8]. Von Maltzahn et al. [9] showed that 120 nm silica core–15 nm gold nanoshells exhibit significantly lower absorption of light compared with nanorods of axial sizes of 12.7 nm and 47 nm, and a narrower bandwidth as well. Meanwhile, Pattani et al. [10] calculated that spherical nanoparticles of 120 nm silica core–7 nm gold shells exhibited higher temperatures than gold nanorods with axial sizes of 25.8 nm and 7.4 nm. The present study is restricted only to spherical nanoparticles.

The GNP size is also a crucial parameter in bio-distribution, circulation in blood, and cellular uptake. Even though nanoparticles with intermediate sizes of 20–60 nm tend to have the highest cellular uptake [11], they also tend to induce systemic toxicity due to injuries in liver, lungs, and spleen [12], compared with smaller sized NPs of 5–10 nm that are highly excreted from renal and hepatobiliary pathways [4,13,14]. Meanwhile, larger sized nanoparticles exhibit larger absorption cross sections and thus a higher LSPR effect, which is shown in the first part of our study. Overall, as Levy et al. [15] concluded, the optimum size for a GNP expressed as the number of particles in a cell might differ when their mass is considered, which further attests to the complexity of GNP size and its effect on bioavailability and cellular uptake.

Different kinds of cell death instigate different immune responses. Apoptotic cancer cells are suggested to stimulate tumor cell repopulation and induce immunological silence or tolerance [16]. In contrast, cancer cells in response to certain anticancer treatments, including cytostatic drugs, radiotherapy, or application of heat above 47 °C for up to 10 min, the so-called “thermal ablation” range, undergo necrosis or necroptosis, that is, immunogenic cell death (ICD) [17]. ICD possesses immunogenic potential, leading to the release of immunostimulatory factors and endogenous molecules, referred to as damage-associated molecular patterns (DAMPs), and to the extracellular milieu, capable of priming an effective cancer-specific immune response, thereby leading to the destruction of any surviving, therapy-resistant cancer cells and the development of immune memory [18]. This is considered a very important biologically based evolution of cancer therapy, also targeting unavoidably any metastases. This cell debris binds to the cognate receptors of innate immune cells such as dendritic cells (DCs) to stimulate their maturation into professional antigen-presenting cells (APCs) and consequently activates CD4^+^ CD8^+^ T lymphocytes by DC-mediated antigen presentation. In addition, ICD is associated with increased infiltration of lymph nodes by B cells, which produce tumor-specific antibodies [19]. Hyperthermia also inhibits DNA repair, including proper processing and amendment of double-strand breaks (DSBs), making it a potent radio- and chemosensitizer, for various types of cancer, including tumors of head and neck, bladder, breast, and cervix [18].

However, cells utilize an evolutionarily conserved defense mechanism that renders them thermotolerant, the so-called heat shock response, where molecular chaperones such as heat shock proteins (HSPs) bind to client proteins denatured by heating in order to mediate their proper folding, their transport into organelles, or their proteosomal degradation [20]. For example, it has been shown that HSP90 inhibitor Ganetespib enhances the cytotoxic effects of hyperthermia, with or without radio- or chemotherapy, and decreases thermotolerance in cervix cancer cell lines [18]. At the transcriptoanal level, heat-activated heat shock factor 1 (HSF1) in mammals induces the expression of genes encoding HSPs (e.g., HSP70 and HSP90) [21]. Of importance, extracellular or membrane bound HSPs (e.g., HSP90) can also act as DAMPs, without contributing to the immunogenic potential of necrotic cancer cells though [22].

In the first part of our study, we investigated the effect of the size, shape, and structure of the nanoparticles on their absorption efficiency for the optical spectrum. This simulation involves the calculation of the absorption cross section of spherical particles and nanoshells using Mie’s theory [19]. The nanoparticles under investigation have spherical, with diameters ranging from 10 nm to 1000 nm, and ellipsoid shapes, and their structure involves gold (bulk), silica cores with gold in the outer layer, and golden cores with a TiO_2_ outer layer.

In the second part of our study, we present a simulation framework of tumor response during hyperthermia treatment mediated by lasers and Au-NPs, and hyperthermia induced by microwaves without the contribution of the nanoparticles. The primary goal of this simulation study was to obtain the thermal profiles of the two types of spherical tumors and estimate the level of tumor shrinkage. In the first form of thermal therapy (i.e., laser-induced hyperthermia with Au-NPs), we predicted the optimal radiation wavelength based on nanoparticles’ selected sizes and material type. We then computed the thermal profile both in the tumorous and the surrounding healthy tissue for a given nanoparticle distribution in the tissue. In the second form of therapy, we investigated the thermal distribution produced by an antenna tuned in varying microwave frequencies. By using a three-state mathematical model of hyperthermic cell death [23], an exponential tumor growth model [24] and calibration of models against experimental data, we obtained a long-term evolution of tumor size for melanoma and prostate cancer. In addition, by our simulation approach, we predict an enhanced tumor shrinkage upon HSP90 inhibition, as also indicated by experimental evidence. The tumors are considered spherical with a radius approximately 3 mm.

## 2. Materials and Methods

### 2.1. Effect of the Size, Shape, and Structure of the Nanoparticles on Their Absorption Efficiency

In this section, we performed a detailed study on the dependence of the absorption efficiency of nanoparticles on their size, shape, and structure. The absorption and scattering cross section of a spherical particle can be calculated using Mie’s theory [19], which has also been applied in the case of nanoshells [25], i.e., nanoparticles in the form of two concentric spheres with different materials in the inner and outer layer. According to this theory, the absorption efficiency of a nanoshell, namely the ratio of the absorption cross section to the geometrical cross section is given by the formula

(1) Qabs=2x22∑n=1∞(2n+1)(Re[an+bn]−|an|2−|bn|2)

where

(2) $$a_{n}=\frac{\psi_{n}\left(x_{2}\right)\left[\psi_{n}^{\prime }\left(m_{2}x_{2}\right)-A_{n}\chi_{n}^{\prime }\left(m_{2}x_{2}\right)\right]-m_{2}\psi_{n}^{\prime }\left(x_{2}\right)\left[\psi_{n}\left(m_{2}x_{2}\right)-A_{n}\chi_{n}\left(m_{2}x_{2}\right)\right]}{\xi_{n}\left(x_{2}\right)\left[\psi_{n}^{\prime }\left(m_{2}x_{2}\right)-A_{n}\chi_{n}^{\prime }\left(m_{2}x_{2}\right)\right]-m_{2}\xi_{n}^{\prime }\left(x_{2}\right)\left[\psi_{n}\left(m_{2}x_{2}\right)-A_{n}\chi_{n}\left(m_{2}x_{2}\right)\right]}$$

(3) $$b_{n}=\frac{m_{2}\psi_{n}\left(x_{2}\right)\left[\psi_{n}^{\prime }\left(m_{2}x_{2}\right)-B_{n}\chi_{n}^{\prime }\left(m_{2}x_{2}\right)\right]-\psi_{n}^{\prime }\left(x_{2}\right)\left[\psi_{n}\left(m_{2}x_{2}\right)-B_{n}\chi_{n}\left(m_{2}x_{2}\right)\right]}{m_{2}\xi_{n}\left(x_{2}\right)\left[\psi_{n}^{\prime }\left(m_{2}x_{2}\right)-B_{n}\chi_{n}^{\prime }\left(m_{2}x_{2}\right)\right]-\xi_{n}^{\prime }\left(x_{2}\right)\left[\psi_{n}\left(m_{2}x_{2}\right)-B_{n}\chi_{n}\left(m_{2}x_{2}\right)\right]}$$

and

(4) $$A_{n}=\frac{m_{2}\psi_{n}\left(m_{2}x_{1}\right)\psi_{n}^{\prime }\left(m_{1}x_{1}\right)-m_{1}\psi_{n}^{\prime }\left(m_{2}x_{1}\right)\psi_{n}\left(m_{1}x_{1}\right)}{m_{2}\chi_{n}\left(m_{2}x_{1}\right)\psi_{n}^{\prime }\left(m_{1}x_{1}\right)-m_{1}\chi_{n}^{\prime }\left(m_{2}x_{1}\right)\psi_{n}\left(m_{1}x_{1}\right)}$$

(5) $$B_{n}=\frac{m_{2}\psi_{n}\left(m_{1}x_{1}\right)\psi_{n}^{\prime }\left(m_{2}x_{1}\right)-m_{1}\psi_{n}^{\prime }\left(m_{1}x_{1}\right)\psi_{n}\left(m_{2}x_{1}\right)}{m_{2}\chi_{n}^{\prime }\left(m_{2}x_{1}\right)\psi_{n}\left(m_{1}x_{1}\right)-m_{1}\chi_{n}\left(m_{2}x_{1}\right)\psi_{n}^{\prime }\left(m_{1}x_{1}\right)}$$

Here, $m_{1}=n_{1}/n_{m}$, $m_{2}=n_{2}/n_{m}$, $x_{1}=2\pi R_{1}n_{m}/\lambda$, $x_{2}=2\pi R_{2}n_{m}/\lambda$ and $\psi_{n}\left(\rho \right)=\rho j_{n}\left(\rho \right)$, $\chi_{n}\left(\rho \right)=-\rho y_{n}\left(\rho \right)$, $\xi_{n}\left(\rho \right)=\rho h_{n}^{\left(1\right)}\left(\rho \right)$, where $n_{1},n_{2},n_{m}$ are the complex refractive indices of the inner layer, the outer layer and the surrounding medium respectively, $R_{1},R_{2}$ are the radii of the inner and outer layer respectively, λ is the wavelength of the incident radiation in vacuum and $j_{n},y_{n},h_{n}^{\left(1\right)}$ are the spherical Bessel functions of the first, second and third kind respectively. These equations can be easily simplified to the case of a single layer spherical nanoparticle setting $A_{n}=B_{n}=0$.

Further, the dielectric constant ε(ω) of small metal particles should be modified in order to consider the scattering of free electrons on the surface of the nanoparticle. Thus, it takes the form [26]

(6) $$\varepsilon \left(\omega ,L_{eff}\right)=\varepsilon_{bulk}\left(\omega \right)+\frac{\omega_{p}^{2}}{\omega^{2}+i\omega \upsilon_{F}/L_{\infty }}-\frac{\omega_{p}^{2}}{\omega^{2}+i\omega \left(\upsilon_{F}/L_{\infty }+A\upsilon_{F}/L_{eff}\right)}$$

where ω is the angular frequency of the incident radiation, $L_{eff}$ is the reduced mean free path length of free electrons, $\varepsilon_{bulk}\left(\omega \right)$ the dielectric constant of the bulk material, $\omega_{p}$ the plasma angular frequency, $\upsilon_{F}$ the Fermi velocity, $L_{\infty }$ the mean free path length of free electrons, and A a dimensionless constant which is usually assumed to be close to unity. The values of these constants for gold are usually taken as [27] $\omega_{p}=1.37\times 10^{16}$ rad/s, $\upsilon_{F}=1.4\times 10^{6}$ m/s, $L_{\infty }=4.2\times 10^{-8}$ m, and A=1. On the other hand, $L_{eff}$ is set equal to the thickness of the gold layer. The dielectric constants of the bulk materials as well as the surrounding medium are taken through a reliable online database [28].

**Figure 1:**
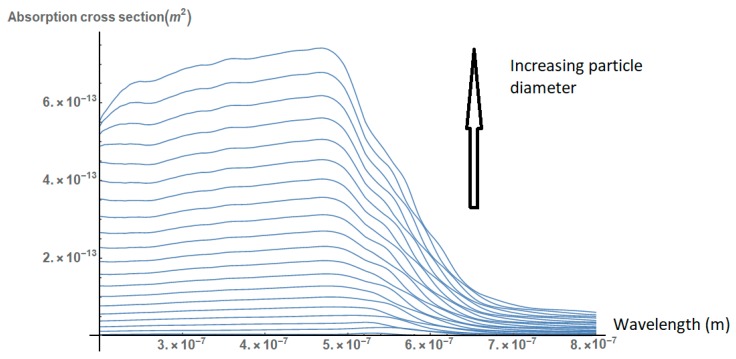
Absorption spectra of gold nanoparticles of different diameters (10–1000 nm). The cross section increases, but the peak lies in the region of 500 nm for all curves.

First, we consider the effect of the particle size on its absorption cross section. As shown in Figure 1, the absorption cross section of the nanoparticles increases relatively to their diameter. However, the peak of the absorption cross section does not change significantly and remains in the region of 500 nm.

**Figure 2:**
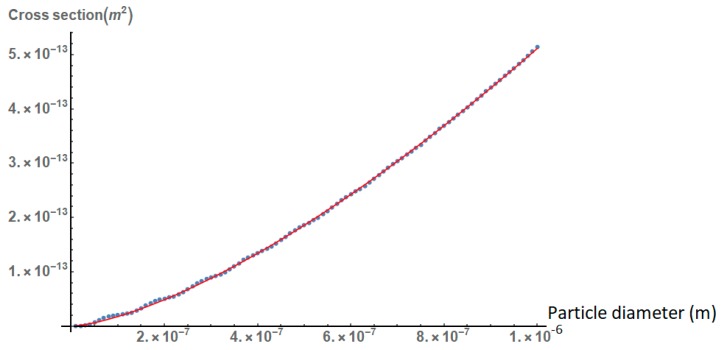
The absorption cross section of gold nanoparticles as a function of their diameter. The red line is a fitting curve based on the function *y* = *αx^p^*, where *α* = 2.9 × 10^−4^ and *p* = 1.46. The incident wavelength is assumed to be 532 nm, which is a common laser wavelength corresponding to the second harmonic of Nd:YAG lasers and is also in the region of maximum absorption of gold nanoparticles.

From Figure 2, it is deduced that the absorption cross section of the gold nanoparticles increases inversely with their diameter $\sim d^{P}$, where the exponent p is of the order of 1.5. This finding shows that, for larger nanoparticles, the absorption cross section does not increase as rapidly with their size as in the case of small nanoparticles, where according to the dipole approximation, the absorption cross section increases $\sim d^{3}$.

**Figure 3:**
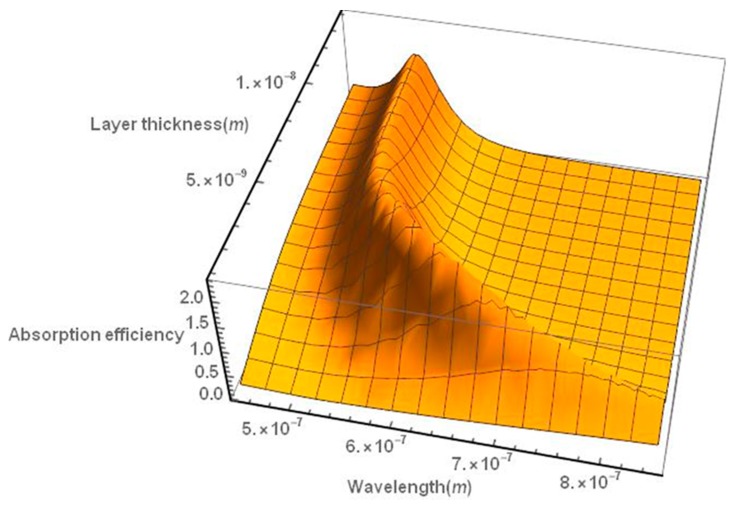
Absorption efficiency spectrum of a gold nanoshell as a function of the thickness of the gold layer. The spectrum is red-shifted as the nanoshell thickness decreases. The particle diameter is assumed to be 30 nm, as in the case of the hyperthermia simulations.

We have also studied the behavior of the absorption efficiency of a nanoshell consisting of a silica core surrounded by a gold layer, as in the case of the hyperthermia simulations. Τhe absorption spectrum of the nanoshell appears to be red-shifted as the thickness of the gold layer decreases (Figure 3).

**Figure 4:**
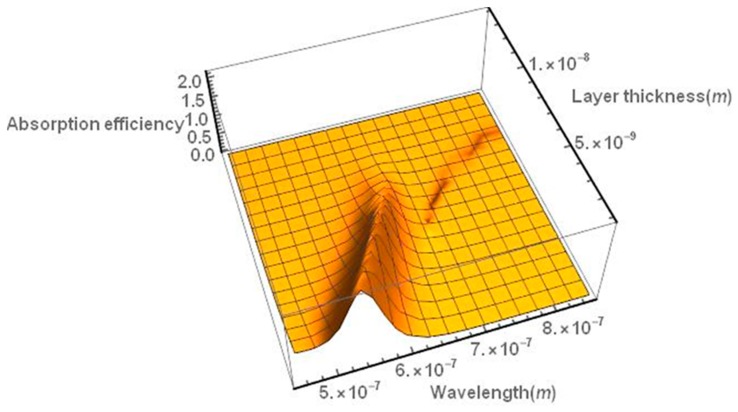
Absorption efficiency spectrum of a gold nanoparticle covered with a TiO_2_ layer as a function of the thickness of the titanium layer. The spectrum is red-shifted as the thickness of the dielectric layer increases. The particle diameter is assumed to be 30 nm.

The absorption spectrum can also be red-shifted by covering the gold nanoparticle with an appropriate layer of dielectric material (e.g., a TiO_2_ layer), as shown in Figure 4.

Another way to red-shift the absorption spectrum of gold nanoparticles is by modifying their shape. Specifically, according to Gann’s theory [29,30,31], the absorption cross section of a non-spherical nanoparticle having the form of a prolonged ellipsoid is given by the formula

(7) $$\gamma_{abs}=\frac{2\pi V\varepsilon_{m}^{3/2}}{3\lambda }\sum_{j}^{}\frac{\left(1/P_{j}^{2}\right)\varepsilon_{2}}{{\left(\varepsilon_{1}+\frac{1-P_{j}}{P_{j}}\varepsilon_{m}\right)}^{2}+\varepsilon_{2}^{2}}$$

where V is the volume of the nanoparticle, $\varepsilon_{m}$ the dielectric constant of the surrounding medium, λ the wavelength of the incident radiation in vacuum, and $\varepsilon_{1},\varepsilon_{2}$ the real and imaginary part of the dielectric constant of the nanoparticle respectively. The parameters $P_{j}$, often referred as depolarization factors, are given by the relations

(8) $$P_{A}=\frac{1-e^{2}}{e^{2}}\left[\frac{1}{2e}\ln\left(\frac{1+e}{1-e}\right)-1\right]$$

(9) $$P_{B}=P_{C}=\frac{1-P_{A}}{2}$$

where

(10) $$e=\sqrt{1-\frac{1}{r^{2}}},\;r=\frac{\ell_{A}}{\ell_{B}}$$

**Figure 5:**
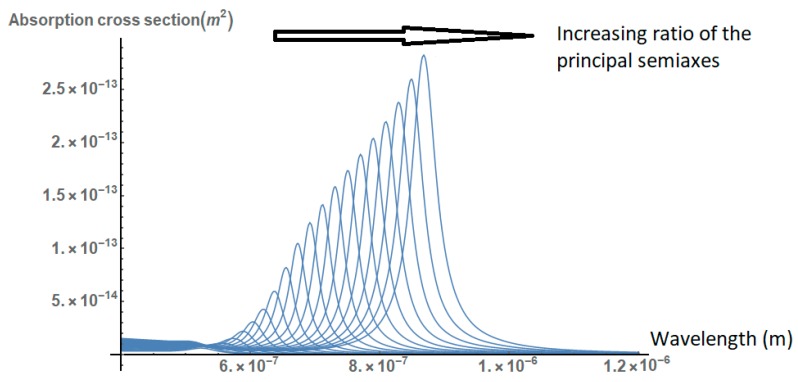
Absorption cross section of a gold nanoparticle in the form of a prolonged ellipsoid, for different values of the ratio of the principal semi axes (1≤r≤5). The cross section is increased and red-shifted as the ratio of the principal axes increases.

Here $\ell_{A}>\ell_{B}=\ell_{C}$ are the lengths of the principal semiaxes of the ellipsoid. As shown in Figure 5, the absorption cross section of the nanoparticle increases and red-shifts significantly as the ratio of the principal semiaxes increases.

Based on the above analysis, the absorption spectrum of the nanoparticles can be tailored to meet the needs of specific applications, either by using a layered structure or by altering the shape of the nanoparticles.

### 2.2. Simulations for Nanoparticle and Microwave-Induced Hyperthermia and Hyperthermic Cell Death

**Figure 6:**
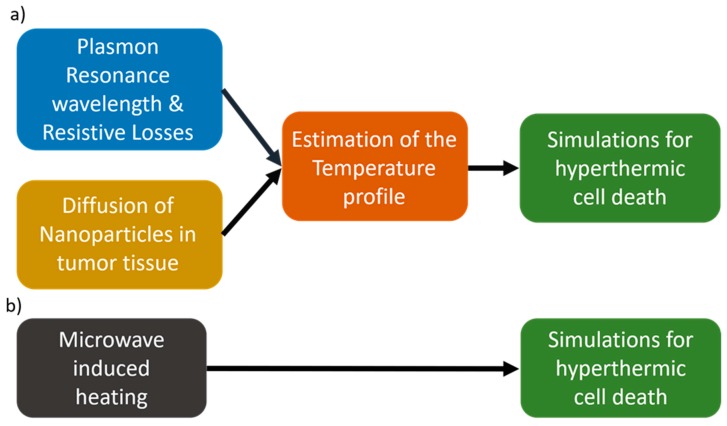
Workflow for the estimation of tumor shrinkage using (**a**) gold nanoparticle-induced hyperthermia and (**b**) using microwave-induced hyperthermia.

In this part, we present a novel study, the aim of which is to calculate the tumor response under hyperthermic conditions. To this end, we tested two methods of thermal treatment. The first method is the nanoparticle-induced hyperthermia and the second method is the traditional microwave-induced hyperthermia. The pipelines that we followed for this study are synopsized in Figure 6a,b.

### 2.3. Nanoparticle- and Microwave-Induced Hyperthermia

**Figure 7:**
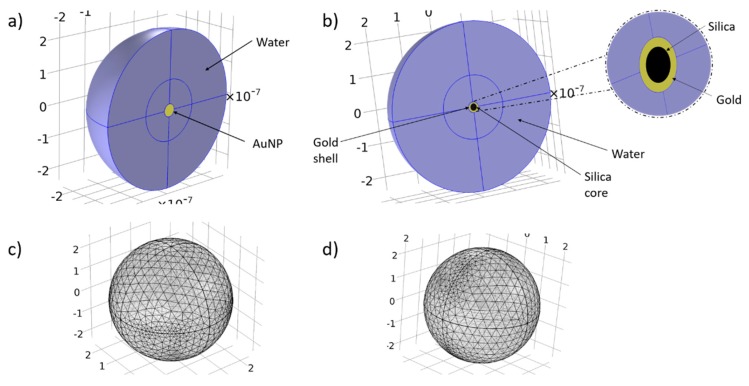
Corresponding geometries and meshes for the simulation of plasmon resonance. (**a**,**c**), AuNP with a 40 nm diameter surrounded by 225 nm of water; (**b**,**d**) AuSiO_2_NP with a 30 nm diameter (20 nm SiO_2_, 5 nm Au layer) surrounded by 225 nm of water.

The first part of the study is focused on the prediction of the thermal profile inside the tumor. The simulations have been developed and formulated with Comsol 5.2 simulation software environment (www.comsol.com). The simulation is divided broadly into two major steps. The first part involves the search of the optimum wavelength in which the heat absorption by nanoparticles is maximized. Two types of nanoparticles were investigated. The first type is a spherical gold nanoparticle with a diameter of 40 nm (Figure 7a,c). The second type of nanoparticle has a silica core with a diameter of 20 nm and is coated with a 5 nm thick gold layer (Figure 7b,d). The density power of the incoming laser beam was set at 20 W/cm^2^. The beam is considered as a continuous wave (CW), and the incoming wave is plane. Water was set as the surrounding environment for both types of nanoparticles. The optical properties of gold were retrieved from Rakic [32], and the ones of silica from the built-in library of Comsol.

(11) $$\nabla \times \left(\nabla \times E\right)-k_{0}^{2}\varepsilon_{r}E=0.$$

The scattering boundary condition on the surface of nanoparticles are defined from the equation:

(12) $$n\times \left(\nabla \times \left(E+E_{b}\right)\right)-\left(jk+1/r\right)n\times \left(E\times n\right)=0.$$

Equation (11) describes the scattering of an incoming plane electromagnetic field, $E\left(r,\varphi ,z\right)=\tilde{E}\left(r,z\right)e^{-im\varphi }$, on the surface of a nanoparticle, and $\varepsilon_{r}={\left(n^{\prime }-jn^{\prime \prime }\right)}^{2}$ is the complex valued relative permittivity calculated from the refractive index $n=n^{\prime }-jn^{\prime \prime }$ of each material, *k*_0_ is the free space wavenumber. Finally, in Equation (12), n is the vector perpendicular to the surface of the nanoparticle, and $E_{b}=Ee^{-ik_{0}x}$ is the background electric field.

In the second part, the laser radiation was applied in the tumor region, where a solution of nanoparticles was injected and diffused in the tissue, and the thermal profile was obtained. The equations that were used for simulation are the following:

(13) $$\frac{\partial c_{i}}{\partial t}+\;\nabla \cdot \left(-D_{i}\nabla c_{i}\right)=R_{i}$$

(14) $$\rho C_{p}u\nabla T+\;\nabla q=Q+\;Q_{bio}$$

(15) $$Q_{bio}=\rho_{b}C_{p,b}\omega_{b}\left(T_{b}-T\right)+Q_{met}.$$

Moreover, Equation (13) describes the diffusion of the injected nanoparticles solution inside the tumorous tissue, where *c_i_* is the variable of concentration of the nanoparticles, *D_i_* is the diffusion coefficient of each material, and *R_i_* is a generation term that was set at 30 nanoparticles/m^3^. The concentration of NPs injected into the tumor is 40 μg/mL [33]. The solution is injected into the center of the tumor. The last equations, Equations (14) and (15), represent the diffusion of the heat produced by the nanoparticles, inside the tumor and the surrounding region, where q=−k∇T (*k* = the thermal conductivity), *u* is the normal vector, *ρ* and *ρ_b_* are the densities of each tissue and of the blood, respectively, *C_p_*, and *C_p,b_* are the specific heat capacities of tissues and blood, *T* and *T_b_* are the temperatures of tissue and arterial blood, *ω_b_* is the blood perfusion rate, and *Q_met_* is the metabolic heat source. The values of the various parameters are presented in Table 1.

**Figure 8:**
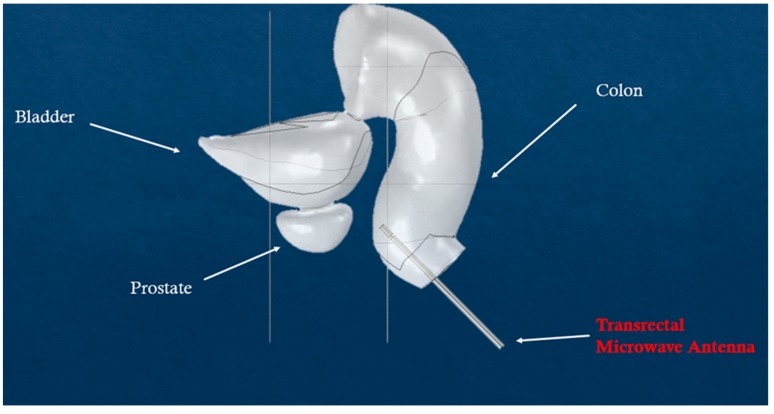
Simulated scenario of microwave-induced hyperthermia for prostate cancer.

**Figure 9:**
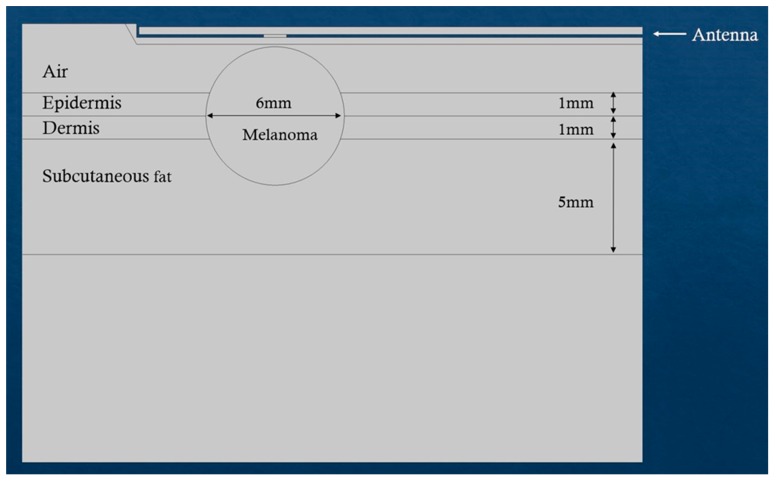
Simulated scenario of microwave-induced hyperthermia for melanoma.

The results were also compared with microwave-induced hyperthermia for both melanoma and prostate cancer. Here, we used the same theoretical background for electromagnetic scattering, but different geometries that include the transrectal microwave antenna for the prostate (Figure 8) and an antenna that is in contact with the skin for the melanoma (Figure 9). Both antennas are operating in 433 MHz and 2.4 GHz.

For the corresponding geometries, fine-sized, free tetrahedral meshes for the nanoparticles, the details of which are shown in Table 2 and Figure 7c,d, and normal-sized, free triangular meshes for the melanoma and prostate tumor geometries were selected and are shown in Table 3.

### 2.4. Estimation of Hyperthermic Cell Death

The second part of the study concerns the prediction of the tumor response and, specifically, the tumor size during the days the patient received laser-induced hyperthermia treatment with Au-NPs. In this part, the models that were used are (i) a three-state mathematical model of hyperthermic cell death [23] and (ii) an exponential model of tumor growth [24]. The first model predicted the shrinkage of the tumor due to hyperthermia treatment and the second one the growth of the tumor because of the survived cells and their subsequent growing and recovering. This last effect is consistent with the idea of cancer recurrence. Recurrence occurs mainly because this “insignificant” number of cancer cells that survived the treatment is too small to be detected in follow-up tests, and unfortunately, over time, these cells start to grow into tumors or cancer.

The prediction of hyperthermic cell death is thoroughly described in a study by O’Neil et al. [23], where they present a three-state mathematical model of hyperthermic cell death in which the “three-state” term stands for the (i) alive, (ii) vulnerable, and (iii) dead states of the cell. The benefits of using this model is its flexibility of describing the cell death due to necrosis (fast cell death) and apoptosis (slow-programmed cell death). The equations that are used are the following:

(16) A+V+D=1

(17) $$\frac{dA}{dt}=-k_{f}A+k_{b}\left(1-A-D\right)$$

(18) $$\frac{dD}{dt}=k_{f}\left(1-A-D\right)$$

(19) $$\frac{dD}{dt}=k_{s}\left(1-D\right).$$

In the first equation (Equation (16)), the three states of the cells are taken into consideration; *A* for alive, *V* for vulnerable, and *D* for dead. Equations (17) and (18) describe the rates of the alive and dead cells during fast cell death. The parameter $k_{f}=\bar{k_{f}}e^{\frac{T}{T_{k}}}\left(1-A\right)$ is a forward rate parameter, where $\bar{k_{f}}$ is a scaling constant, *T* is the temperature variable, $T_{k}$ sets the rate of exponential increase according to temperature, and $k_{b}$ is a backward rate constant. The last equation, Equation (19), describes the rate changes of dead cells during slow cell death, where $k_{s}=\bar{k_{s}}D\left(1-D\right){\left(D-D_{\tau }\right)}^{2}$. The constant $\bar{k_{s}}$ is a baseline scaling value for *k_s_*, and *D_τ_* is a threshold value of maximum cell death for cultures that have suffered minimal thermal damage. The parameters $\bar{k_{f}}$, $k_{b}$, $T_{k}$, $k_{s}$, and $D_{\tau }$ were estimated using the function “fmincon,” which finds the minimum of constrained nonlinear multivariable functions, provided by MATLAB; experimental data for melanoma were obtained from Blanco-Andujar et al. [39] and Feng et al. [40], and for prostate cancer were derived from Huang et al. [41]. The optimized parameters are presented in Table 4. In slow cell death, the cells can be considered either as “dead” or “not dead.” As a result, in slow cell death, there is not a vulnerable phase, and $k_{f}=k_{b}=0$.

### 2.5. Tumor Growth Model

Besides the cell death model, the growth of the tumor was modeled with a simple exponential growth [24], when the effect of the treatment was over. The equation is the following:

(20) $$\frac{dV}{dt}=a_{0}V.$$

Here, the coefficient $a_{0}$ was estimated from experimental data. The simple exponential model was calibrated against experimental data from Proia et al. [42] for melanoma and Gao et al. [43] for prostate cancer (Table 5). Proia et al. also investigated the melanoma growth differentiation upon HSP90 inhibition (Figure 11).

## 3. Results

**Figure 10:**
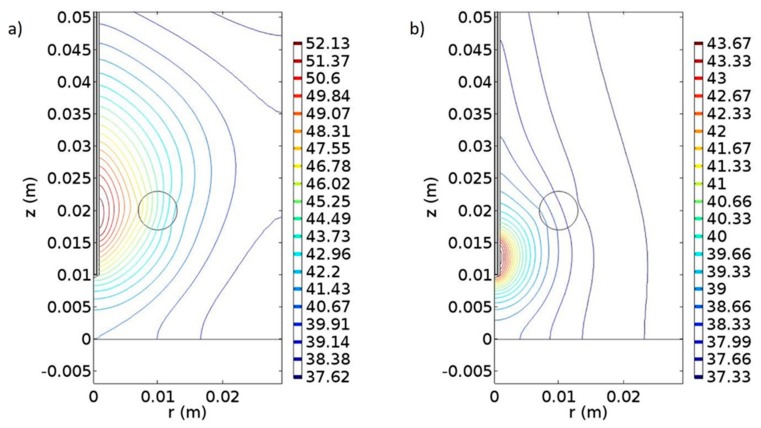
Simulation of microwave-induced temperature effects on prostate tissue. (**a**) Temperature distribution at the 9th min of treatment where the antenna is tuned at 2.4 GHz with power of 10 W. (**b**) Temperature distribution at the 30th min of heating where the antenna was tuned at 433 MHz with power of 30 W. The unit of the contours is in Celsius degrees, and the unit of x- and y-axes in meters.

From the first simulation study, we observed in Figure 10a the wavelengths in which the LSPR effect occurred. The plasmon peak for the Au-SiNP and AuNP was found at 590 and 540 nm, respectively. Experimental data show that the plasmon peak for AuNPs, with a diameter approximately 40 nm, can be found at 535 nm [44], and Sambou et al. [45] showed that for the (20 nm diameter) silica-core/(5 nm thickness) Au-NPs it can be found at around 600 nm. Moreover, we simulated the diffusion of nanoparticles inside the tumorous tissue. The tumor was considered spherical with a radius of approximately 3 mm and a volume of 113 mm^3^. In the diffusion study, the NPs diffuse radially, following a Gaussian form, outwards both to the tumorous (r ≤ 3 mm) and the surrounding healthy tissue (r > 3 mm), forming a concentration gradient (Figure 12b). The distribution of the NPs in the cancerous region has taken place in such a way that the induced thermal effect would not damage the healthy tissue. Concluding the first part of our study, we have produced thermal profiles of the tumorous and the surrounding healthy tissue at the 10th minute of the heating procedure (Figure 12c). The intratumoral temperature surpasses the threshold of cell damage, while the temperature of the surrounding healthy tissue, between 3 and 6 mm, is raised to a degree that can cause damage.

The temperature generated by the nanoparticle-based therapy appears to be sufficient for the eradication of the corresponding tumors. The effect of the traditional microwave-induced hyperthermia for the melanoma and prostate tumor, respectively, are shown in Figure 13 and Figure 10. In the microwave-based therapy, the increased temperature was distributed in a much larger area compared to the NP-generated temperature. Apparently, combining hyperthermia with NPs resulted in an enhanced therapeutic effect probably because of the tumor-targeted delivery of NPs. Microwave ablation enables rapid heating, which leads to coagulative necrosis [46]; however, this heat is not limited to the tumorous tissue but propagates outwards into adjacent tissue [47].

Generally, the antitumor effectiveness of thermotherapy is dependent on the temperature and duration of therapy [48]. Specifically, in the melanoma when the antenna is tuned at 2.4 GHz at 10 W (Figure 13a), the temperature can activate apoptotic mechanisms at the 9th minute of the therapy. When the antenna is set at 433 MHz and 100 W (Figure 13b) at the 30th minute, the temperature is high enough to induce necrosis in the cells. Our findings also support the “dogmatic notion” that apoptosis is not the preferred form of cell death, given that apoptotic cells do not possess such a high immunostimulatory potential. Membrane rupture and release of DAMPs, like in the case of necrotic cells, is required to prime immunity [16,49].

In the prostate cancer, we followed the same procedures as in the case of melanoma. When the antenna was set at 2.4 GHz and 10 W, the temperature that is generated is capable of inducing apoptotic mechanisms at the 9th minute of therapy (Figure 10a), and when the antenna was set at 433 MHz and 30 W, cell necrosis can begin at the 30th minute (Figure 10b). Comparing the two frequencies, 2.4 GHz and 433 MHz, differences in the distribution of the temperature in the space could be observed.

Regarding the second simulation case study, we present the tumor evolution over time for two cases: (i) prostate cancer and (ii) melanoma. Both tumor volumes are set at 113 mm^3^. The temperature for the hyperthermic cell death of the prostate tumor is set at 50 °C and 48 °C for the melanoma. The use of the three-state model helps us observe the fast cell death during the first 30 min of heating for the melanoma and 15 min (Figure 14a) prostate cancer (Figure 14c). The reduced viability due to the slow apoptotic cell death for the next 48 h after treatment is shown in Figure 14b–d. From the cell death and the tumor growth model, we obtained the evolution of tumor size during time (Figure 15). The results depicted in Figure 15 indicate the reduction of tumor size when the patient receives therapy every 4 days for seven sessions in the Melanoma case, and on the 0th, 2nd, and 6th days in the Prostate case. In the first two days of each 4-day interval, the cells were damaged and underwent apoptotic cell death. In the next two days, the cancer cells recovered and started to proliferate again. This resulted in a slight increase in the tumor volume after the hyperthermic treatment. Therefore, tumor cells appear to develop resistance to thermotherapy, as in the case of chemoradiotherapy [50,51], leading to tumor regrowth. In a clinical setting, this can contribute to cancer’s recurrence and relapse [50].

**Figure 11:**
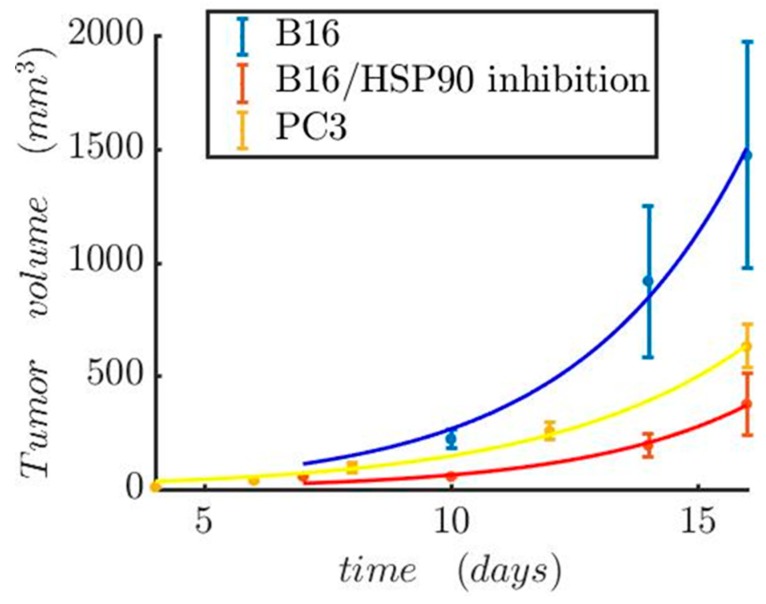
Tumor growth model. Prostate tumor growth in the absence of therapy and melanoma growth patterns both in the presence and absence of HSP90. The experimental data were taken from Proia et al. [42] for melanoma and Gao et al. [43] for prostate cancer.

**Figure 12:**
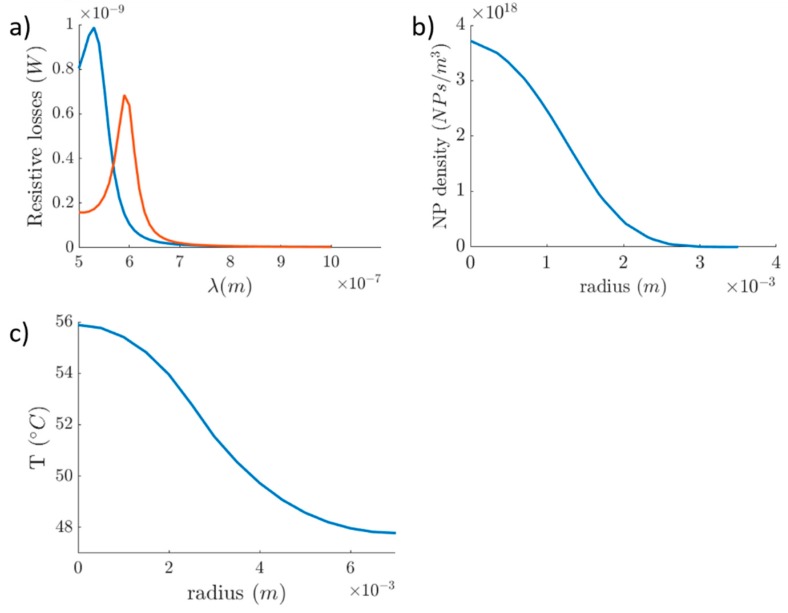
Resonant wavelength of two different size nanoparticles. (**a**) The blue line corresponds to the 40 nm AuNP and the orange line to the 30 nm Au-SiNP. (**b**) Distribution of NPs inside the tumorous tissue 1 h after the injection in the center of the tumor. (**c**) Temperature rise during the first 10 min of NP laser-induced heating.

**Figure 13:**
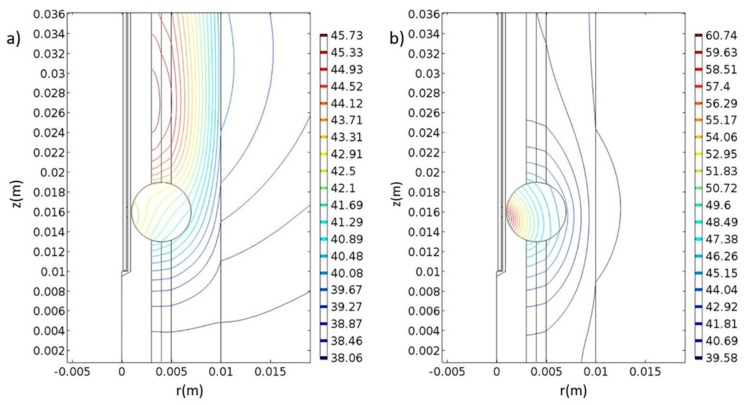
Simulation of microwave-induced temperature effects for melanoma tissue. (**a**) Temperature distribution at the 9th minute of treatment where the antenna is tuned at 2.4 GHz with power of 10 W. (**b**) Temperature distribution at the 30th minute of heating and the antenna is tuned at 433 MHz with 100 W power. The unit of the contours in Celsius degrees, and the unit of x- and y-axes in meters.

**Figure 14:**
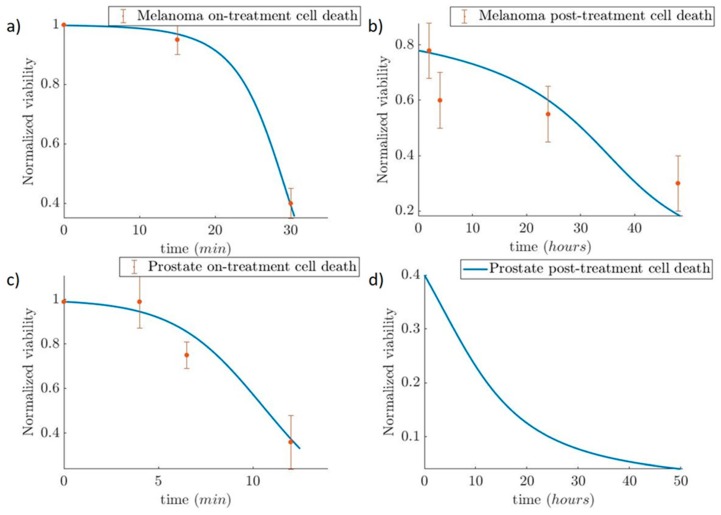
Simulation of tumor response to hyperthermia treatment. (**a**,**c**) Depiction of the fast cell death that occurs during the treatment for melanoma and the prostate cancer cells. (**b**,**d**) Depiction of post-treatment slow cell death for melanoma and the prostate cancer cell, respectively. The experimental data have been taken from Blanco-Andujar et al. [39] and Feng et al. [40] for melanoma, and Huang et al. [41] for prostate cancer.

**Figure 15:**
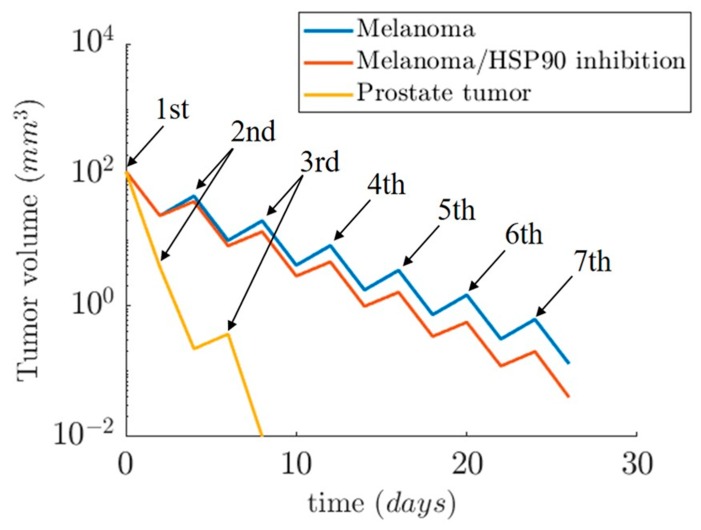
Estimated results for tumor shrinkage upon hyperthermia complete treatment (3–7 sessions) for two cancer types. The therapeutic sessions for Melanoma were repeated every 4 days. In the Prostate tumor, the time interval between the first two sessions was 2 days, while the interval between the 2nd and 3rd session was 4 days. For Melanoma, the expected values considering HSP90 inhibition were included.

Of particular importance, inhibition of HSP90 is associated with increased tumor shrinkage (Figure 11 and Figure 15), which could be translated to increased overall survival in cancer patients [50]. This is probably due to the “thermoresistance” potential of HSP90 [18]. Moreover, the interacting patterns of HSP90 (HSP90AA1) were investigated with the usage of STRING v10.5 [52], a comprehensive database of protein associations derived from diverse sources. A highly interconnected HSP90-mediated network was generated (Figure S1). HSP90 interacts with several fellow HSPs and other molecular chaperones, including DNAJB1, AHSA1/2, CDC37, and CDC37L1. HSP also appears to be associated with cancer-relevant factors, such as the hypoxia-inducible factor HIF1A, the prominent tumor suppressor TP53, CDK1, SRC, MYC, NOS3, VEGFA, EGFR, ERBB2, MAPK1, AKT1/2, and RPS6KB1. This suggests that HSP90 and the HSP90-interacting molecules are functionally associated to confer resistance to heat-treated tumors. At the same time, these data open the possibility for better hyperthermia-based tumor treatment when for example combined with siRNA or other molecular targeting of not only HSPs but also another closely associated protein, as shown in Figure S1.

## 4. Discussion

Simulations provide useful tools in cancer therapy based on their ability to visualize and predict the phenomena that occur during cancer treatment (e.g., radiation effects), further enabling therapy optimization, in order to be as effective as possible and to minimize the side effects in adjacent normal tissues. Microwave-induced hyperthermia is governed by physical mechanisms and effects that can be easily modeled because of the existing knowledge on electromagnetism and heat transfer effects.

In this simulation study, we present a hyperthermia treatment model mediated by Au-NPs, which includes a prediction of the thermal effect on tumor evolution, i.e. estimation of tumor shrinkage over time with the usage of mathematical models. We have also performed simulation of microwave-induced temperature effects for melanoma and prostate tissue. In this case, we have demonstrated the presence of quantitative and qualitative differences between different types of tissues, but with significant increases in temperatures above 50–60 °C in some tissue areas. We have deduced from this current study that depending on the energy and frequency used, these temperature effects can be modulated in order to target specific tumor regions. We also provide insights for a possible creation of a model for the role of HSP90 protein and potentially other HSPs and closely associating proteins revealed by the use of bioinformatics. In the case of prostate tumors, we observed a much higher potency of HSP90 inhibition toward thermosensitization. Interestingly, in a recent work, the knockdown of HSP70 and HSP90 was efficiently exploited by the use of gold nanorods loaded with HSP inhibitor-VER-155008 micelles for the destruction of colon cancer cells mediated by mild-temperature photothermal therapy [53].

Our findings revealed that the nanoparticle-induced hyperthermia has a more localized thermal effect, in the tumor tissue, as compared to the microwave-induced hyperthermia in the given frequencies. This result was expected, because the thermal profile is strongly correlated with the distribution and the density of the nanoparticles in the tumor tissue. On the other hand, differences in penetration depth among the two frequencies were observed. Microwave-induced hyperthermia, as it is currently applied, lacks the ability of precise heat localization compared to the nanoparticle-induced hyperthermia. Therefore, its application in the clinic can be improved by the implantation of iron oxide nanoparticles that can enhance the localized heat effect in tumor tissue.

The findings of the present study could be exploited for the design of anti-neoplastic therapeutic strategies, where radiation/NP-induced thermotherapy and simultaneous pharmacologic inhibition of molecular chaperones (HSPs) could be utilized to both sensitize resistant cancer cells to death and increase their immunological potential. For example, hypoxia-related studies have shown that in breast cancer cells the uptake of NPs was increased in hypoxic microenvironments, as compared to normoxic conditions in head and neck squamous cell carcinoma (HNSCC) cells [54].

## Figures and Tables

**Table 1:** Selected parameters for blood, dermis, epidermis, fat, tumor, and muscles tissues, as used in Equations (2)–(4).

	Specific Heat Capacity Cp (J/kg/°C)	Density *ρ* (kg/m³)	Thermal Conductivity k (W/m∙°C)	Blood Perfusion Rate w (m³/m³∙s)	Metabolic Heat Source qm (W/kg)	Diffusivity m^2^/s
Blood	3617 [34]	1050 [34]	0.52 [34]	-	1090 [35]	-
Dermis	3300 [36]	1200 [36]	0.45 [36]	1.25 × 10^−3^ [36]	1200 [37]	-
Epidermis	3590 [36]	1200 [36]	0.23 [36]	0 [36]	1200 [37]	6.2 × 10^−11^ [38]
Fat	2348 [34]	911 [34]	0.21 [34]	1.25 × 10^−3^ [36]	464 [37]	-
Tumor/Muscle	3421 [34]	1090 [34]	0.49 [34]	1.65 × 10^−3^ [34]	991 [34]	

**Table 2:** Parameters of the meshes for the corresponding geometries of nanoparticles.

	AuNP 20 nm (Surrounded by 225 nm of Water) (Figure 7a,c)	AuSiO_2_NP 30 nm (Surrounded by 225 nm of Water) (Figure 7b,d)
Max element size	39.2 nm	38.4 nm
Min element size	4.9 nm	4.8 nm
Max element growth rate	1.45	1.45
Curvature factor	0.5	0.5
Resolution of narrow regions	0.6	0.6

**Table 3:** Parameters of the meshes for the corresponding geometries of melanoma and prostate tumors.

	Melanoma (Figure 9)	Prostate (Figure 10)
Max element size	5.36 mm	5.36 mm
Min element size	24 μm	24.5 μm
Max element growth rate	1.3	1.3
Curvature factor	0.3	0.3
Resolution of narrow regions	1	1

**Table 4:** Parameters for the hyperthermic cell death model.

	Melanoma	Prostate Cancer
Temperature (degrees Celsius)	48	50
$\bar{k_{f}}$ (min^−1^)	0.25481	0.18946
$k_{b}$ (min^−1^)	0.66477	0.23063
$T_{k}$ (degrees Celsius)	40.1513	39.678
$\bar{k_{s}}$ (hours^−1^)	0.59547	$0.316\times 10^{-3}$ (no data)
$D_{\tau }$	$0.45003\times 10^{-3}$	0.208 (no data)

**Table 5:** Calibrated parameters for the tumor growth model.

	Melanoma	Melanoma (HSP90 Inhibited)	Prostate Cancer
α_0_ (s^−1^)	0.328 ± 0.003	0.237 ± 0.005	0.243 ± 0.016

## Supplementary Materials

The following are available online at http://www.mdpi.com/2079-4991/9/2/167/s1. Figure S1. HSP90 interactome; confidence score of interactions more than 90%. The nodes represent genes/proteins and the lines indicate connection between nodes. The molecular action visualization mode was used.

## References

[1] Pavlopoulou A., Bagos P.G., Koutsandrea V., Georgakilas A.G. (2017). Molecular determinants of radiosensitivity in normal and tumor tissue: A bioinformatic approach. Cancer Lett..

[2] Prasad V. (2017). Tisagenlecleucel—The first approved CAR-T-cell therapy: Implications for payers and policy makers. Nat. Rev. Clin. Oncol..

[3] Dewhirst M.W., Vujaskovic Z., Jones E., Thrall D. (2005). Re-setting the biologic rationale for thermal therapy. Int. J. Hyperth..

[4] Dimitriou N.M., Tsekenis G., Balanikas E.C. (2017). Gold nanoparticles, radiations and the immune system: Current insights into the physical mechanisms and the biological interactions of this new alliance towards cancer therapy. Pharmacol. Ther..

[5] Cherukuri P., Glazer E.S., Curley S.A. (2010). Targeted hyperthermia using metal nanoparticles. Adv. Drug Deliv. Rev..

[6] Keisari Y. (2017). Tumor abolition and antitumor immunostimulation by physico-chemical tumor ablation. Front. Biosci..

[7] Van de Broek B., Devoogdt N., D’Hollander A., Gijs H.L., Jans K., Lagae L., Muyldermans S., Maes G., Borghs G. (2011). Specific cell targeting with nanobody conjugated branched gold nanoparticles for photothermal therapy. ACS Nano.

[8] Liu H., Liu T., Wu X., Li L., Tan L., Chen D., Tang F. (2012). Targeting gold nanoshells on silica nanorattles: A drug cocktail to fight breast tumors via a single irradiation with near-infrared laser light. Adv. Mater..

[9] von Maltzahn G., Park J.H., Agrawal A., Bandaru N.K., Das S.K., Sailor M.J., Bhatia S.N. (2009). Computationally guided photothermal tumor therapy using long-circulating gold nanorod antennas. Cancer Res..

[10] Pattani V.P., Tunnell J.W. (2012). Nanoparticle-mediated photothermal therapy: A comparative study of heating for different particle types. Lasers Surg. Med..

[11] He C., Hu Y., Yin L., Tang C., Yin C. (2010). Effects of particle size and surface charge on cellular uptake and biodistribution of polymeric nanoparticles. Biomaterials.

[12] Vinluan R.D., Zheng J. (2015). Serum protein adsorption and excretion pathways of metal nanoparticles. Nanomedicine.

[13] Armanetti P., Pocoví-Martínez S., Flori A., Avigo C., Cassano D., Menichetti L., Voliani V. (2018). Dual photoacoustic/ultrasound multi-parametric imaging from passion fruit-like nano-architectures. Nanomed. Nanotechnol. Boil. Med..

[14] Cassano D., Summa M., Pocoví-Martínez S., Mapanao A.-K., Catelani T., Bertorelli R., Voliani V. (2018). Biodegradable Ultrasmall-in-Nano Gold Architectures: Mid-Period In Vivo Distribution and Excretion Assessment. Part. Part. Syst. Charact..

[15] Levy R., Shaheen U., Cesbron Y., See V. (2010). Gold nanoparticles delivery in mammalian live cells: A critical review. Nano Rev..

[16] Jondal D.E., Thompson S.M., Butters K.A., Knudsen B.E., Anderson J.L., Carter R.E., Roberts L.R., Callstrom M.R., Woodrum D.A. (2018). Heat Stress and Hepatic Laser Thermal Ablation Induce Hepatocellular Carcinoma Growth: Role of PI3K/mTOR/AKT Signaling. Radiology.

[17] Soni S., Tyagi H., Taylor R.A., Kumar A. (2014). Investigation on nanoparticle distribution for thermal ablation of a tumour subjected to nanoparticle assisted thermal therapy. J. Therm. Boil..

[18] Vriend L.E.M., van den Tempel N., Oei A.L., L’Acosta M., Pieterson F.J., Franken N.A.P., Kanaar R., Krawczyk P.M. (2017). Boosting the effects of hyperthermia-based anticancer treatments by HSP90 inhibition. Oncotarget.

[19] Mie G. (1908). Beiträge zur Optik trüber Medien, speziell kolloidaler Metallösungen (Contributions to the optics of turbid media, especially colloidal metal suspensions). Ann. Phys..

[20] Ellis R.J., Hartl F.U. (1999). Principles of protein folding in the cellular environment. Curr. Opin. Struct. Boil..

[21] Calderwood S.K., Murshid A. (2017). Molecular Chaperone Accumulation in Cancer and Decrease in Alzheimer’s Disease: The Potential Roles of HSF1. Front. Neurosci..

[22] Kroemer G., Galluzzi L., Kepp O., Zitvogel L. (2013). Immunogenic cell death in cancer therapy. Annu. Rev. Immunol..

[23] O’Neill D.P., Peng T., Stiegler P., Mayrhauser U., Koestenbauer S., Tscheliessnigg K., Payne S.J. (2011). A Three-State Mathematical Model of Hyperthermic Cell Death. Ann. Biomed. Eng..

[24] Benzekry S., Lamont C., Beheshti A., Tracz A., Ebos J.M.L., Hlatky L., Hahnfeldt P. (2014). Classical Mathematical Models for Description and Prediction of Experimental Tumor Growth. PLoS Comput. Boil..

[25] Tuersun P., Han X.E. (2013). Optical absorption analysis and optimization of gold nanoshells. Appl. Opt..

[26] Keribig U., Vollmer M. (2015). Optical Properties of Metal Clusters.

[27] Averitt R.D., Westcott S.L., Halas N.J. (1999). Linear optical properties of gold nanoshells. J. Opt. Soc. Am. B.

[28] https://refractiveindex.info/.

[29] Gans R. (1915). Über die Form ultramikroskopischer Silberteilchen. Ann. Phys..

[30] Papavassiliou G.C. (1979). Optical properties of small inorganic and organic metal particles. Prog. Solid State Chem..

[31] Link S., Mohamed M.B., El-Sayed M.A. (1999). Simulation of the Optical Absorption Spectra of Gold Nanorods as a Function of Their Aspect Ratio and the Effect of the Medium Dielectric Constant. J. Phys. Chem. B.

[32] Rakić A.D., Djurišić A.B., Elazar J.M., Majewski M.L. (1998). Optical properties of metallic films for vertical-cavity optoelectronic devices. Appl. Opt..

[33] Qian L.P., Zhou L.H., Too H.-P., Chow G.-M. (2011). Gold decorated NaYF_4_:Yb,Er/NaYF_4_/silica (core/shell/shell) upconversion nanoparticles for photothermal destruction of BE(2)-C neuroblastoma cells. J. Nanopart. Res..

[34] Ali M.Y., Grimm C.F., Ritter M., Mohr L., Allgaier H.-P., Weth R., Bocher W.O., Endrulat K., Blum H.E., Geissler M. (2005). Activation of dendritic cells by local ablation of hepatocellular carcinoma. J. Hepatol..

[35] Rai N.K., Rai K.S. (1999). Effect of metabolic heat generation and blood perfusion on the heat transfer in the tissues with a blood vessel. Heat Mass Transf..

[36] Gowrishankar T.R., Stewart D.A., Martin G.T., Weaver J.C. (2004). Transport lattice models of heat transport in skin with spatially heterogeneous, temperature-dependent perfusion. Biomed. Eng. Online.

[37] Jiao J., Guo Z. (2009). Thermal interaction of short-pulsed laser focused beams with skin tissues. Phys. Med. Boil..

[38] Cornelissen L.H., Bronneberg D., Oomens C.W.J., Baaijens F.P.T. (2008). Diffusion measurements in epidermal tissues with fluorescent recovery after photobleaching. Skin Res. Technol..

[39] Blanco-Andujar C., Ortega D., Southern P., Nesbitt S.A., Thanh N.T.K., Pankhurst Q.A. (2015). Real-time tracking of delayed-onset cellular apoptosis induced by intracellular magnetic hyperthermia. Nanomedicine.

[40] Feng Y., Oden J.T., Rylander M.N. (2008). A Two-State Cell Damage Model Under Hyperthermic Conditions: Theory and In Vitro Experiments. J. Biomech. Eng..

[41] Huang H.-C., Rege K., Heys J.J. (2010). Spatiotemporal Temperature Distribution and Cancer Cell Death in Response to Extracellular Hyperthermia Induced by Gold Nanorods. ACS Nano.

[42] Proia D.A., Kaufmann G.F. (2015). Targeting Heat-Shock Protein 90 (HSP90) as a Complementary Strategy to Immune Checkpoint Blockade for Cancer Therapy. Cancer Immunol. Res..

[43] Gao F., Al-Azayzih A., Somanath P.R. (2015). Discrete functions of GSK3α and GSK3β isoforms in prostate tumor growth and micrometastasis. Oncotarget.

[44] Zuber A., Purdey M., Schartner E., Forbes C., van der Hoek B., Giles D., Abell A., Monro T., Ebendorff-Heidepriem H. (2016). Detection of gold nanoparticles with different sizes using absorption and fluorescence based method. Sens. Actuators B Chem..

[45] Sambou A., Ngom B.D., Gomis L., Beye A.C. (2016). Turnability of the Plasmonic Response of the Gold Nanoparticles in Infrared Region. Am. J. Nanomater..

[46] Brace C.L. (2010). Microwave tissue ablation: Biophysics, technology, and applications. Crit. Rev. Biomed. Eng..

[47] Saldanha D.F., Khiatani V.L., Carrillo T.C., Yap F.Y., Bui J.T., Knuttinen M.G., Owens C.A., Gaba R.C. (2010). Current tumor ablation technologies: Basic science and device review. Semin. Interv. Radiol..

[48] Zhu Q., Zhang A., Liu P., Xu L.X. (2012). Study of tumor growth under hyperthermia condition. Comput. Math. Methods Med..

[49] Tait S.W., Ichim G., Green D.R. (2014). Die another way--non-apoptotic mechanisms of cell death. J. Cell Sci..

[50] Nikolaou M., Pavlopoulou A., Georgakilas A.G., Kyrodimos E. (2018). The challenge of drug resistance in cancer treatment: A current overview. Clin. Exp. Metastasis.

[51] Pavlopoulou A., Oktay Y., Vougas K., Louka M., Vorgias C.E., Georgakilas A.G. (2016). Determinants of resistance to chemotherapy and ionizing radiation in breast cancer stem cells. Cancer Lett..

[52] Szklarczyk D., Morris J.H., Cook H., Kuhn M., Wyder S., Simonovic M., Santos A., Doncheva N.T., Roth A., Bork P. (2017). The STRING database in 2017: Quality-controlled protein-protein association networks, made broadly accessible. Nucleic Acids Res..

[53] Tang X., Tan L., Shi K., Peng J., Xiao Y., Li W., Chen L., Yang Q., Qian Z. (2018). Gold nanorods together with HSP inhibitor-VER-155008 micelles for colon cancer mild-temperature photothermal therapy. Acta Pharm. Sin. B.

[54] Chen E.Y., Hodge S., Tai K., Hou H., Khan N., Hoopes P.J., Samkoe K.S. (2013). Oxygen microenvironment affects the uptake of nanoparticles in head and neck tumor cells. Proc. SPIE Int. Soc. Opt. Eng..

