# Supplementary material for: Prediction of Gold Nanoparticle and Microwave-Induced Hyperthermia Effects on Tumor Control via a Simulation Approach
Source: Nanomaterials (Basel). 2019 Jan 29;9(2):167. doi: 10.3390/nano9020167 (PMC6410344; doi:10.3390/nano9020167)
Supplement: Supplementary file 1 [file nanomaterials-09-00167-s001.pdf]

Prediction of Gold Nanoparticle and Microwave-Induced Hyperthermia Effects on Tumor Control via a Simulation Approach

Nikolaos M. Dimitriou [1,2], Athanasia Pavlopoulou [3], Ioanna Tremi [1], Vassilis Kouloulias [4], Georgios Tsigaridas [1], and Alexandros G. Georgakilas [1,*]

[1] Department of Physics, School of Applied Mathematical and Physical Sciences, National Technical University of Athens (NTUA), Zografou Campus, 15780 Athens, Greece. nikolaos.dimitriou@mail.mcgill.ca (N.M.D.); ioannatremi@mail.ntua.gr (I.T.); gtsig@mail.ntua.gr (G.T.)

[2] Department of Bioengineering, McGill University, Montreal, H3A 0E9, Quebec, Canada

[3] Izmir International Biomedicine and Genome Institute, Dokuz Eylül University, 35340, Turkey; athanasia.pavlopoulou@deu.edu.tr

[4] Radiation Oncology Unit, 2nd Department of Radiology, Attikon University General Hospital, Medical School, National and Kapodistrian University of Athens, Athens, Greece; vkouloul@ece.ntua.gr

* Correspondence: alexg@mail.ntua.gr

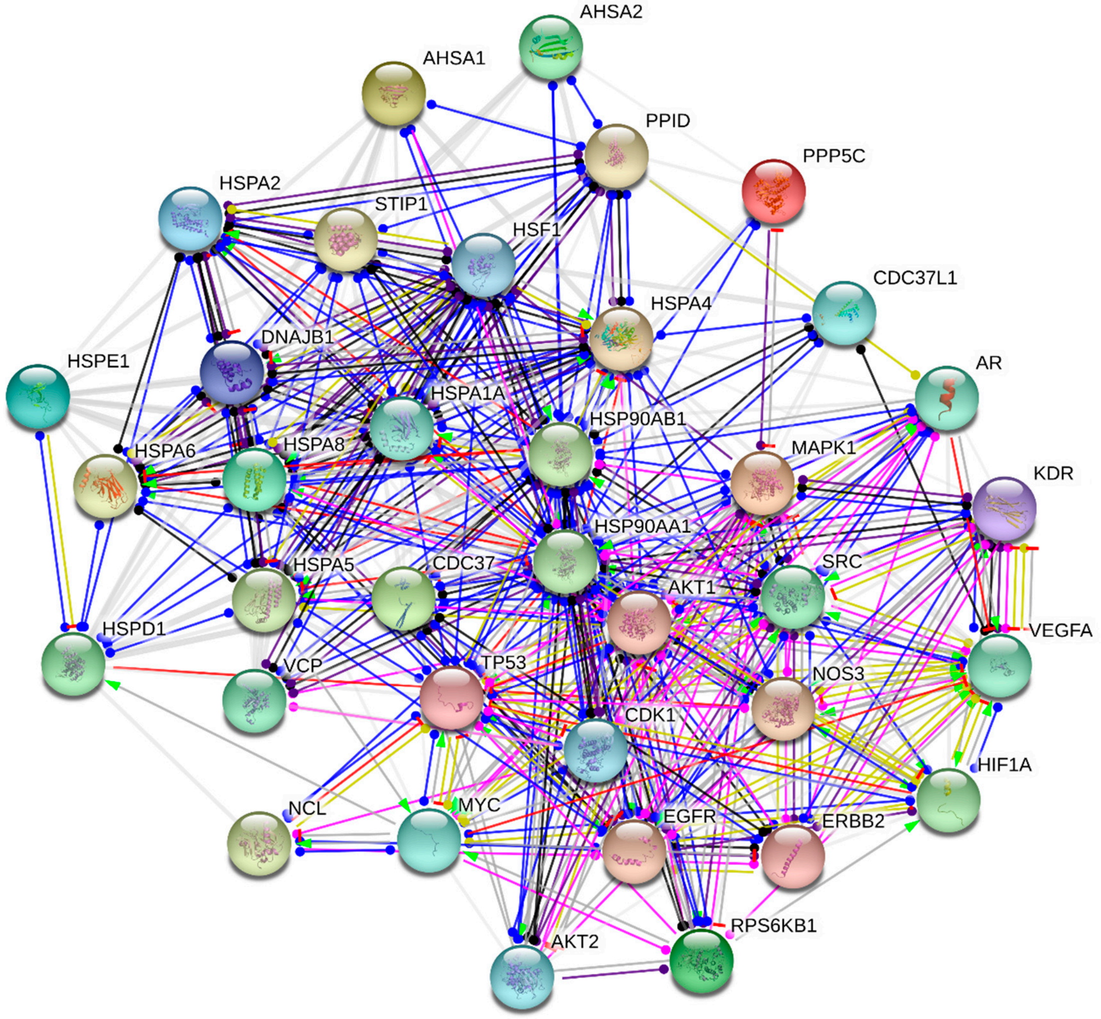

Figure S1. HSP90 interactome; confidence score of interactions more than 90%. The nodes represent genes/proteins and the lines indicate connection between nodes. The molecular action visualization mode was used.
